# Graft incorporation and stem subsidence in femoral impaction bone grafting for revision hip arthroplasty: a systematic review and meta–analysis of 2514 hips

**DOI:** 10.1007/s00402-025-06122-1

**Published:** 2025-11-12

**Authors:** Artsiom Klimko, Octavian Andronic, Victor Yan Zhe Lu, Dimitris Dimitriou, Armando Hoch, Patrick O. Zingg

**Affiliations:** 1 University Hospital Balgrist, Zurich, Switzerland; 2 The Chinese University of Hong Kong, Hong Kong, China

**Keywords:** Femoral impaction bone grafting, Revision hip arthroplasty, Graft incorporation, Stem subsidence, Bone loss, Cortical repair, Trabecular incorporation, Trabecular remodeling

## Abstract

**Background:**

Femoral impaction bone grafting (IBG) is an established technique for managing severe bone loss during revision total hip arthroplasty (rTHA). Despite its widespread use, the extent of graft incorporation and the degree of stem subsidence remain incompletely characterized. This systematic review evaluates graft incorporation and stem subsidence outcomes in femoral IBG for rTHA.

**Methods:**

A systematic review was conducted following the PRISMA (Preferred Reporting Items for Systematic reviews and Meta–Analyses) guidelines. Three databases were searched from inception to December 31, 2024, for studies involving rTHA with femoral IBG and a minimum follow–up of 12 months. Meta–analyses focused on graft incorporation rates and stem subsidence. Subgroup analysis examined age, pre–operative bone loss, graft type, follow–up duration and other predictors. Heterogeneity was assessed using the I^2^ statistic under a random–effects model.

**Results:**

A total of 33 studies (2395 patients; 2514 hips) met inclusion criteria. The mean patient age was 66 years (range 22–95 years), with a male–to–female distribution of 48%/52%. The hips–weighted mean follow–up was 8.8 years (range of study means 1.1–17.0 years). Overall, the pooled proportion of graft incorporation was 76%; 95% confidence interval (CI) 63%–85%. The weighted average subsidence across all studies was 2.5 mm (95% CI 1.7–3.1 mm). Subgroup analyses showed no statistically significant differences in graft incorporation rates based on graft type (*p* = 0.399), age (*p* = 0.742), or follow–up duration (*p* = 0.560). Similarly, stem subsidence did not differ significantly by gender (*p* = 0.181), graft type (*p* = 0.460), or age (*p* = 0.301). Preoperative bone loss classification (Endo–Klinik, Paprosky) was not associated with notable differences in graft incorporation (*p* = 0.263) or subsidence (*p* = 0.486).

**Conclusions:**

Femoral IBG for rTHA demonstrates variable but generally favorable graft incorporation rates, averaging 76% (95% CI 63%–85%), with a mean stem subsidence of 2.5 mm at mid–to–long–term follow–up. Neither graft type, stem design, age, nor preoperative bone loss classification significantly influenced subsidence or incorporation variability.

## Introduction

Revision total hip arthroplasty (rTHA) often presents a significant challenge in the setting of compromised femoral bone stock and severe bone loss [11]. Femoral impaction bone grafting (IBG) remains a widely used technique for restoring bone stock and achieving biological integration of graft material in such cases [8, 36]. Despite its clinical utility, concerns persist regarding graft integration and stem subsidence, both of which can lead to early construct failure [27, 50].

The extent of stem subsidence can be influenced by multiple factors, including stem design (e.g., polished, tapered stems vs. matte or collared designs), graft type, surgical technique, and patient–specific variables such as age, gender, and the severity of bone loss [3, 50]. Reported rates of stem subsidence vary greatly (20%–94.30%), with some studies indicating considerable axial migration that remains asymptomatic [7, 53], while others associate subsidence with eventual implant failure [8, 26]. In instances of severe bone loss, achieving adequate graft impaction and stem stability may necessitate additional fixation strategies [41].

Insufficient or slow graft integration can also lead to progressive stem migration and ultimately, to aseptic loosening [13]. Therefore, it is critical to identify the key determinants of graft incorporation and stem stability to optimize outcomes. This systematic review consolidates existing evidence on stem subsidence and graft integration in femoral IBG during rTHA, with the aim of clarifying the most influential factors and guiding improved surgical decision–making for long–term implant stability.

## Materials and methods

### Strategy of the systematic search

This systematic review was conducted following the Preferred Reporting Items for Systematic Reviews and Meta–Analyses (PRISMA) guidelines [31] and was registered in the International Prospective Register for Systematic Reviews (PROSPERO) under registration number CRD42024557047. A comprehensive search was carried out across Cochrane Central Register of Controlled Trials (CENTRAL), MEDLINE (via PubMed) and EMBASE for studies published up until December 31, 2024. The search focused on studies related to femoral IBG in rTHA. Keywords used in the search strategy included combinations of the following terms: “impaction bone grafting,” “revision hip arthroplasty,” “femoral reconstruction,” “hip stem revision,” “femoral bone loss,” and “graft incorporation.” Included studies involved patients undergoing rTHA with femoral IBG with a minimum follow–up period of 12 mon.

### Selection process and data extraction

Two authors (AK and VL) independently and in duplicate screened study titles and abstracts—those that met the inclusion criteria underwent full text evaluation. Any disagreements between reviewers were resolved through consultation with a third author (OA). The inclusion criteria were: i) Published peer–reviewed human studies in English; ii) A minimum level of evidence of IV based on the Oxford Centre for Evidence–Based Medicine 2011 Levels of Evidence (reference); iii) Studies that reported outcomes related to stem subsidence and/or graft incorporation rates following femoral IBG in rTHA. The exclusion criteria included: i) Review articles, hypothesis or technique papers, or oral presentations; ii) Non–English language publications; iii) Cadaveric or animal studies; iv) Studies with overlapping patient populations; v) Case reports or series with fewer than 10 patients; vi) Studies involving primary total hip arthroplasty (THA), acetabular defects, or benign femoral lesions; vii) Animal or pre–clinical studies; viii) Technical or cost–effectiveness studies; ix) Studies reporting short–term follow–up (< 1 year); x) Studies without a DOI; xi) Revisions in patients with rheumatoid or genetic pathologies; xii) Studies involving cancer patients.

For studies that reported only subsidence ranges without exact values, central tendencies and dispersions were derived using established statistical methods appropriate for incomplete data reporting. Specifically, when authors provided ranges (e.g., minimum, maximum) without accompanying means or standard deviations, the mean estimation formula was employed [17]. Studies with unquantifiable subsidence or significant gaps in radiographic follow–up were excluded. Graft categories were harmonized as follows: fresh–frozen allograft (non–irradiated); processed allografts (irradiated and/or freeze–dried/lyophilized and/or pasteurized); autograft (alone or mixed).

### Risk of bias assessment

The risk of bias in the included studies was evaluated using the Methodological Index for Non–Randomized Studies (MINORS) criteria [40]. This tool assesses eight key aspects of study design for non–comparative studies and an additional four aspects for comparative studies. Each item was scored as 0 if not reported, 1 if reported but inadequate, and 2 if adequately reported, with a maximum score of 16 for non–comparative studies and 24 for comparative studies. Two independent reviewers performed the assessment, with any discrepancies resolved through consensus or by consultation with a senior author.

### Statistical analysis

Quantitative data from the included studies, such as graft incorporation rates, subsidence, re–revision rates, and complication rates, were used for meta–analyses where comparable data were available. Meta–analyses were conducted using the ‘metafor’ package in R (R Core Team, R Foundation for Statistical Computing, Vienna, Austria) [48]. A random–effects model with inverse–variance weighting was applied to pool effect sizes. Graft incorporation rates and stem subsidence were compared across cohorts by calculating standardized mean differences where appropriate. Follow–up is summarized as a hips–weighted mean across studies and, to reflect skewness, as the median of study means with IQR; a true patient–level median was not estimable from study–level data. The primary migration outcome was mean femoral stem subsidence (mm) at the prespecified timepoint; pooling was conducted on the continuous scale. For clinical interpretability, we predefined two descriptive thresholds based on prior IBG literature: clinically relevant subsidence ≥ 5 mm and massive subsidence ≥ 10 mm [7, 8, 14],Ten [44]. Based on prior IBG and radiostereometric analysis (RSA) literature showing front–loaded migration, we defined the first postoperative year as the primary subsidence period [13, 19, 29, 33]. Because a minority of series report small additional settling beyond year 1 and many studies cluster their reporting at 2–3 years, we prespecified a 3–year cutoff for subgrouping (≤ 3 vs > 3 years) to capture any late settling while maintaining comparability across studies.

For binary outcomes such as the occurrence of complications or re–revision rates, pooled proportions were calculated. Heterogeneity was assessed using Higgins and Thompson’s I^2^ statistic and Cochran’s Q test [4, 15]. Given the limitations of these metrics, prediction intervals were also calculated to estimate the range in which the effect size of future studies is expected to fall. Standard deviations were estimated using the Wan et al. estimator [49] or derived from p–values as per the Cochrane Handbook (Higgins JPT). Heterogeneity was classified as moderate when I^2^ exceeded 40% and high when it exceeded 75% [45].

### Subgroup analysis

We conducted a subgroup analysis of bone loss severity using the Paprosky and Endo–Klinik classification systems. In both systems, Grades I and II were grouped as moderate bone loss, and Grades III and IV were classified as severe bone loss. This approach reflects the fact that higher–grade defects typically involve more extensive structural compromise and may demand distinct surgical strategies, potentially influencing graft incorporation and stem subsidence. Where studies included subgrades (e.g., Paprosky IIIA vs. IIIB), these were merged into the main grade (e.g., Grade III).

## Results

### Study selection and characteristics

**Fig. 1:**
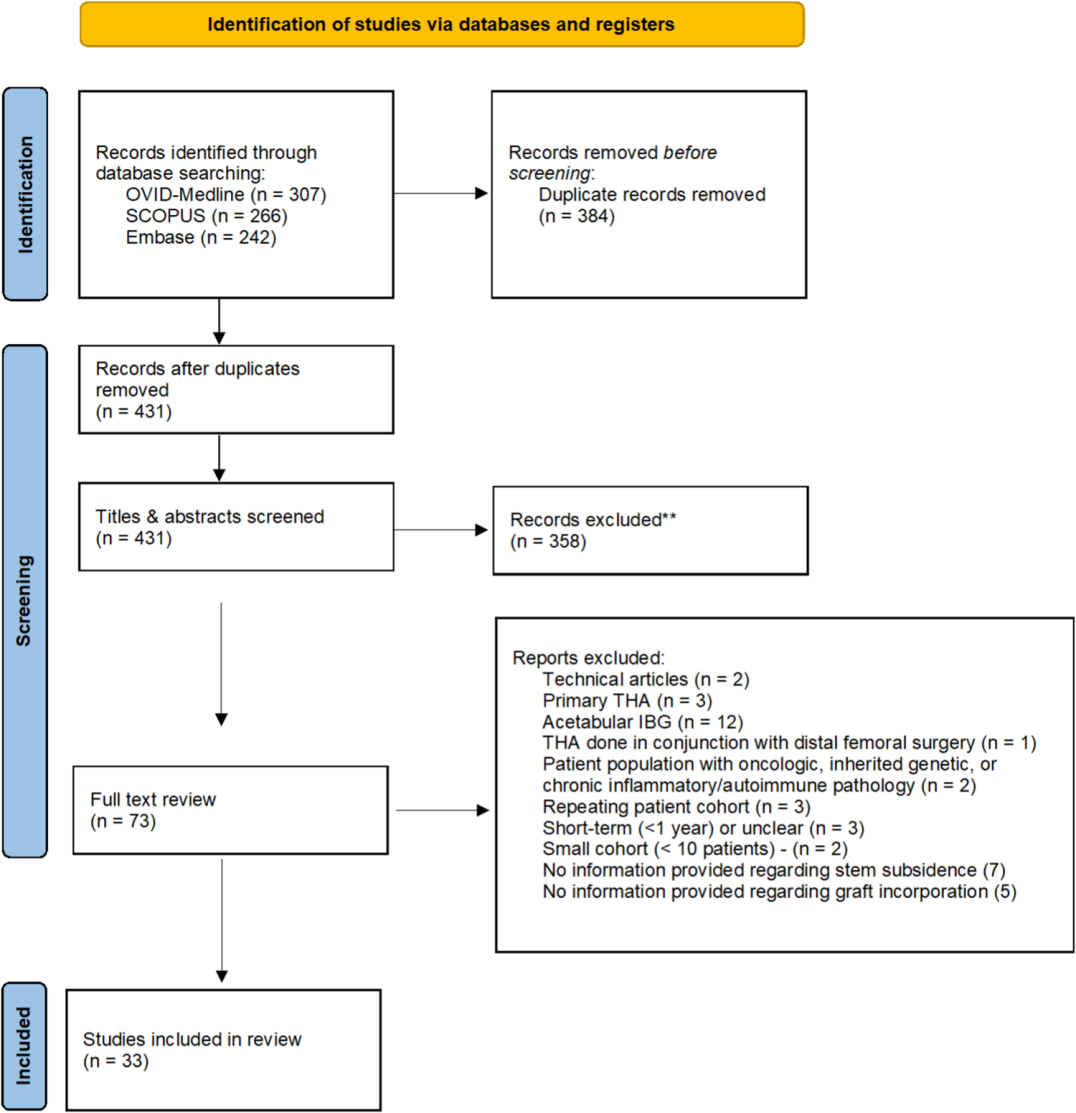
PRISMA flowchart for study selection into systematic review

This systematic review included data from 33 studies, encompassing a total of 2395 patients and 2514 hips (Table 1). *The PRISMA flowchart of the systematic search* is presented in Fig. 1. The mean age of patients included was 66 years (22–95 years), with a 48%/52% male/female distribution. The hips–weighted mean follow–up was 8.8 years (range of study means 1.1–17.0 years); the median of study means was 6.5 years (IQR 3.6–10.1). Indications for revision surgery were primarily driven by aseptic loosening, which was the leading cause in 82% of studies (27/33). Periprosthetic fractures were the second most common indication, reported in 42% of studies (14/33).

**Table 1:** Overview of included studies

Author and year	MINORs score	Numbers of hips in study	Age (mean, range)	Gender (Male/Female)	Follow–up (mean, range)	Indications for revision	Bone Graft Type (e.g., fresh–frozen, autograft, allograft)	What type and concentration/dosage of antibiotics was mixed with bone graft or cement?	Preoperative Bone Loss Classification	Stem/Femoral Component Length (Standard (150 mm), Long (range 200 mm to 260 mm) or Short (125 mm))	Reason for re–revision failure (number of hips)
Bunting et al. [3]	14/16–87.5%	29	61 (43–84)	21.0/8.0	7.4 (0.6–13.9)	Infection	Autograft	Vancomycin added to bone graft; cement: Simplex P with tobramycin or Palacos R + G with gentamycin	Paprosky—Grade I: 4, Grade II: 14, Grade IIIA: 8, Grade IIIB: 1, Grade IV: 2	Standard: 130 mm (62%), 180 mm (27.5%), Long: 200 mm, 260 mm	Persistent infection (n = 3)
Iwase et al. [18]	12/16–75%	99	66 (36–84)	21.0/72.0	11.0 (2–23.8)	Aseptic loosening, secondary reconstruction after infection, femoral osteolysis, periprosthetic fracture with aseptic loosening	Fresh frozen femoral head allograft	Not specified	Endo–Klinik classification: Grades I–IV	Not specified	Aseptic acetabular component loosening (n = 1), recurrent dislocation (n = 2), infection (n = 4), periprosthetic femoral fracture (n = 8)
Park et al. [32]	13/16–81.3%	13	50 (22–77)	7.0/6.0	11.1 (5.3–15.1)	Paprosky IV femoral bone defects	Fresh frozen femoral head allograft	Not specified	Paprosky—Grade IV: 4	Not specified	None
Stigbrand et al. [42]	13/16–81.3%	69	69 (49–88)	40.0/29.0	7.0 (0.7–13.2)	Aseptic loosening, septic loosening	Fresh frozen femoral head allograft	Gentamycin in bone cement	Preop Endo–Klinik—Grade II: 7, Grade III: 46, Grade IV: 16	Standard: 170 mm (64 cases), Long: 150 mm (5 cases)	Mechanical failure (n = 3), dislocation (n = 1)
Wilson et al. [50]	14/16–87.5%	705	70 (22–95)	296.0/335.0	14.7 (9.8–28.3)	Aseptic loosening, periprosthetic fracture, infection, malposition, stem fracture, cement–in–cement exchange	Fresh–frozen non–irradiated allograft, irradiated allograft, pasteurized allograft, bone substitute	Teicoplanin (400 mg IV), Gentamycin (3 mg/kg IV), Vancomycin (1 g added to each femoral head)	Not specified	Standard: 150 mm, Long: 200–260 mm, Short: 125 mm	Aseptic loosening (n = 7), periprosthetic fracture (n = 23), infection (n = 24), malposition (n = 1), fracture of the stem (n = 1), cement–in–cement exchange during acetabular revision (n = 19)
Wimmer et al. [51]	19/24–79.2%	243	69 (49–88)	23.0/47.0	4.4 (NA)	Aseptic loosening, periprosthetic fracture, infection, shaft fissure, avulsion of greater trochanter	Fresh frozen femoral head allograft	Not specified	Paprosky—Grade I: 4, Grade IIA: 20, Grade IIB: 15, Grade IIC: 12, Grade III: 19	Not specified	Revisions: study group (n = 6, 8.6%), control group (n = 19, 11%)
Ten Have et al. [44]	13/16–81.3%	31	65 (35–82)	6.0/23.0	12.6 (10.0–14.7)	Aseptic loosening, aseptic loosening with periprosthetic fracture, conversion of Girdlestone excision arthroplasty, mid–thigh pain	Fresh frozen femoral head allograft	Erythromycin and colistin in Simplex cement	Preop Endo–Klinik—Grade I: 1, Grade II: 10, Grade III: 9, Grade IV: 11	Standard: 150 mm	Mechanical failure (subsidence > 15 mm, n = 4), periprosthetic fracture (n = 4)
Howie et al. [16]	20/24–83.3%	56	63 (27–88)	42.0/14.0	NA (2.0–15.0)	Aseptic loosening, infection, periprosthetic fracture, femoral stem fracture	Fresh frozen femoral head allograft	Vancomycin, 0.25 g per femoral head	Endo–Klinik classification: Grades I–IV	Standard and Long–length stems used	Series 1: stem loosening (n = 4), infection (n = 1); Series 2: infection (n = 1); Series 3: None
Sierra et al. [38]	11/16–68.6%	42	74 (49–89)	14.0/26.0	7.5 (NA)	Aseptic loosening, acute periprosthetic fracture, periprosthetic fracture nonunion, infection	Fresh frozen femoral head allograft	Not specified	Endo–Klinik classification: Grade I: 2, Grade II: 4, Grade III: 25, Grade IV: 9	Long: 220–260 mm	Reoperations: aseptic loosening (n = 2), infections (n = 2), femoral fractures (n = 2)
Wraighte and Howard [52]	12/16–75%	75	68 (35–87)	40.0/35.0	10.5 (6.3–14.1)	Aseptic loosening, periprosthetic fracture, deep infection	Morsellized allograft	Not specified	Endo–Klinik classification: Grade I: 2, Grade II: 19, Grade III: 50, Grade IV: 4	Not specified	Revisions: deep infection (n = 1), periprosthetic fracture (n = 1), fracture of femoral component (n = 1), dislocation and aseptic loosening (n = 1)
Ornstein et al. [29]	13/16–81.3%	15	74 (60–82)	7.0/7.0	5.0 (NA)	Aseptic loosening	Fresh frozen femoral head allograft	Gentamycin in cement, Cloxacillin (systemic)	Gustilo and Pasternak: Type I: 7, Type II: 6, Type III: 2	Standard	Not specified
Halliday et al. [13]	11/16–68.6%	226	68 (35–89)	91.0/116.0	8.1 (5.0–16.0)	Aseptic loosening, femoral fractures	Fresh frozen femoral head allograft	Gentamicin–loaded cement	Endo–Klinik classification: Grade I: 12, Grade II: 106, Grade III: 62, Grade IV: 6	Long: 150–260 mm	Not specified
Gore et al. [12]	11/16–68.6%	26	68 (34–89)	9.0/17.0	NA (0.0–6.0)	Degenerative joint disease, avascular necrosis, rheumatoid arthritis, femoral neck fractures with a painful prosthesis	Morselized allograft bone	Not specified	Not specified	Collarless, polished, tapered stem from cobalt chrome alloy	Postoperative periprosthetic fractures requiring surgery (n = not specified)
Lind et al. [23]	13/16–81.3%	87	NA	NA	3.6 (1.0–7.0)	Aseptic loosening (83), septic loosening (4)	Fresh frozen femoral head allograft	Simplex cement with antibiotics	Endo–Klinik classification: Grade I: 7, Grade II: 34, Grade III: 39, Grade IV: 7; Mallory classification: Grade I: 5, Grade II: 30, Grade IIIa: 25, Grade IIIb: 19, Grade IIIc: 8	Not specified	Recurrent dislocations (n = 1), technical failure (n = 1), loosening during acetabular revision (n = 1)
Robinson et al. [35]	10/16–62.5%	57	64 (31–87)	29.0/24.0	2.3 (0.5–5.7)	Aseptic loosening (55), septic loosening (1), periprosthetic fracture (1)	Irradiated femoral heads	Not specified	Endo–Klinik grade 1: 8, grade 2: 22, grade 3: 27, grade 4: 0	Standard length (150 mm shoulder to tip)	Revisions: subsidence and periprosthetic fractures (n = 2)
Biezen et al. [2]	13/16–81.3%	21	65 (33–82)	3.0/18.0	5.0 (3.4–7.1)	Aseptic loosening, femoral fracture, painful porous–coated prosthesis, Girdlestone situation	Fresh frozen femoral head allograft	erythromycin and colistin–soaked Simplex cement	Endo–Klinik classification: Grade III or IV	Standard: 150 mm	No re–revisions
Pekkarinen et al. [34]	10/16–62.5%	68	71 (40–88)	28.0/37.0	3.0 (0.1–6.0)	Aseptic loosening, infected total hip replacement, dislocation of the prosthesis	Fresh frozen femoral head allograft	Not specified	Endo–Klinik classification: Grade I: 8, Grade II: 22, Grade III: 25, Grade IV: 13	Standard	Re–revision: rotational instability (n = 3), fracture of proximal femur (n = 1)
Flugsrud et al. [10]	13/16–81.3%	10	74 (65–82)	5.0/5.0	4.0 (3.0–4.6)	Not specified	Morsellized allograft	Not specified	Paprosky—Grade II: 1, Grade III: 9	Not specified	None
Kärrholm et al. [19]	14/16–87.5%	24	65 (38–84)	10.0/14.0	3.0 (NA)	Loosening of femoral stem	Morsellized allograft	Gentamicin with cement	Gustilo and Pasternak: Type I: 3, Type II: 17, Type III: 4; Endo–Klinik: Grade I: 1, Grade II: 16, Grade III: 7	Standard: 135 mm	None
Eldridge et al. [8]	12/16–75%	86	68 (27–88)	41.0/38.0	1.1 (0.5–2.8)	Aseptic loosening	Impacted cancellous allograft	Not specified	Endo Klinik classification: Grade I: 1, Grade II: 16, Grade III: 7	Not specified	Subsidence and varus positioning (n = 8)
Gie et al. [11]	11/16–68.6%	56	69 (46–87)	32.0/24.0	2.5 (1.5–4.1)	Femoral component loosening, poor bone stock	Fresh frozen femoral head allograft	Antibiotic–loaded cement of reduced viscosity	Endo–Klinik—Grade I: 3, Grade II: 40, Grade III: 13	Standard–length femoral components	Not specified
Deakin and Bannister [6]	12/16–75%	59	69 (NA)	42.0/43.0	3.7 (0.5–11.0)	Aseptic loosening, infection, periprosthetic fracture, femoral stem fracture	Washed irradiated allograft with autologous marrow	Cemented with Palacos R with gentamicin	Endo–Klinik—Grade I: 9, Grade II: 24, Grade III: 26	Standard and long–length stems used	Persistent infection (n = 2), instability (n = 1)
Edwards et al. [7]	10/16–62.5%	70	66 (26–83)	24.0/50.0	3.1 (1.5–5.2)	Aseptic loosening	Fresh frozen femoral head allograft	Not specified	Endoklinik: Grade I (3), Grade II (9), Grade III (19), Grade IV (7)	Not specified	Sepsis (n = 1), graft failure with significant migration (n = 1), acetabular component migration (n = 1)
Knight and Helming [21]	13/16–81.3%	31	70 (42–97)	17.0/14.0	2.6 (0.6–3.8)	Aseptic stem loosening, osteolysis, intractable thigh pain, periprosthetic fracture, loosening of a cemented acetabular component	Fresh frozen femoral head allograft	Not specified	AAOS: Type I/II (24), Type III (1), Type IV (6)	Not specified	Revision: removal of cement fragment from the joint space (n = 1)
Mikhail et al. [28]	12/16–75%	43	59 (47–84)	22.0/18.0	6.0 (5.0–7.0)	Aseptic mechanical failure of cemented and cementless femoral stems	Fresh frozen femoral head allograft	Not specified	Endo–Klinik—Grade II: 13, Grade III: 23, Grade IV: 7	Not specified	Not specified
Oshima et al. [30]	12/16–75%	55	70 (49–88)	12.0/42.0	7.7 (3.0–12.5)	Secondary osteoarthritis (23), Osteonecrosis (5), Femoral neck fracture (25), Rheumatoid arthritis (2)	Frozen morselized allografts and hydroxyapatite	Not specified	Endo–Klinik—Grade I: 3, Grade II: 24, Grade III: 28	Not specified	Not specified
Schreurs et al. [37]	13/16–81.3%	33	63 (33–82)	9.0/24.0	10.4 (8.0–13.0)	Aseptic loosening (26), Septic loosening (7)	Fresh frozen femoral head allograft	0.5 g erythromycin and 3 million units colistin per 41–g packet of cement	Endo–Klinik—Grade I: 3, Grade II: 14, Grade III: 12, Grade IV: 4	Not specified	None
Singh and Bhalodiya [39]	11/16–68.6%	53	59 (44–68)	42.0/6.0	5.5 (NA)	Aseptic osteolysis, periprosthetic fractures, broken femoral stem, septic loosening, traumatic comminuted fracture	Mixture of autograft and allograft	Not specified	Paprosky—Grade IIA: 1, Grade IIIA: 32, Grade IIIB: 5	Long stems (190–385 mm)	None
Park et al. [33]	10/16–62.5%	47	55 (39–75)	37.0/10.0	13.5 (10.9–17.8)	Aseptic loosening (38), septic loosening (5), periprosthetic fractures (4)	Morselized allograft	Antibiotic bone cement (Simplex P, Howmedica) with cephalosporin or vancomycin	Endo–Klinik—Grade II: 12, Grade III: 16, Grade IV: 11	Standard	None
Te Stroet et al. [43]	13/16–81.3%	37	76 (39–93)	17.0/20.0	9.0 (5.0–16.0)	Aseptic loosening (23), septic loosening (5), dislocation (2), chronic pain (2), aseptic loosening with fracture (1), periprosthetic fracture (4)	Fresh–frozen femoral head allograft	Erythromycin (500 mg) and colistin (3,000,000 IU) in Simplex Bone Cement	Endo–Klinik—Grade 2: 11, Grade 3: 20, Grade 4: 6	Long (205 mm—30, 220 mm—4, 240 mm—2, 260 mm—1)	Recurrent dislocation (n = 1)
Verspeek et al. [47]	13/16–81.3%	33	46 (30–61)	14.0/19.0	17.0 (12.0–22.0)	Aseptic loosening (19), septic loosening (14)	Fresh–frozen femoral head allograft	Not specified	Della Valle—Type 1: 7, Type 2: 17, Type 3A: 6, Type 3B: 1, Type 4: 2	Not specified	Recurrent dislocation (n = not specified), aseptic loosening (n = not specified), septic loosening (n = not specified)
Yan et al. [53]	13/16–81.3%	15	61 (38–84)	4.0/9.0	7.7 (4.0–13.0)	Aseptic loosening (14), infection (1)	Fresh–frozen femoral head allograft	Not specified	Endo–Klinik grade 2: 4, grade 3: 7, grade 4: 4	Standard length	Stem revised due to peri–prosthetic fracture (n = 1), no stems radiographically loose

IBG, impaction bone grafting; rTHA, revision total hip arthroplasty; THA, total hip arthroplasty; RSA, radiostereometric analysis; DEXA, dual–energy X–ray absorptiometry; AAOS, American Academy of Orthopaedic Surgeons; CI, confidence interval; I^2^, I–squared statistic

### Graft materials and surgical techniques

Fresh–frozen femoral head allograft was the most commonly used bone graft material, reported in 73% of the studies (24/33). Morselized allograft and autograft were less frequently used, comprising 12% (4/33) and 9% (3/33) of the studies, respectively. Information on bone chip size was reported in 35% of the studies, with graft sizes varying from 2 to 10 mm. However, 42% of studies did not provide specific details on graft particle size (Table 1).

Preoperative bone loss is presented in Table 1 and was reported in 79% of studies (26/33). The Endo–Klinik classification was the most frequently utilized system, reported in approximately 45% (15/33) of studies, followed by the Paprosky classification in 30% (10/33) of the studies. Severe bone loss, corresponding to Grades III and IV, was noted in 60% of cases using the Paprosky system and 55% using the Endo–Klinik classification.

Stem length data were available in 28 of the 33 studies; among these, 19 (58%) reported using standard–length stems (150 mm) and 10 (30%) employed long stems (200–260 mm), typically in cases with significant bone loss (Table 1).

### Re–revisions and complications

Regarding re–revision failures, infection remained a significant contributor, with persistent infection reported as a reason for re–revision in several studies (Table 1). Infections accounted for re–revision in 7 studies, with 24 hips undergoing re–revision for this reason. Periprosthetic fractures and mechanical failure were also frequent causes, contributing to 23 and 4 re–revisions, respectively. Aseptic loosening contributed to re–revision in 7 hips. Other causes included dislocation, stem loosening (unspecified etiologies), subsidence, and technical failures.

### Stem subsidence

Among studies reporting subsidence, the hips–weighted mean follow–up was 8.8 years (range of study means 1.1–17.0 years); the weighted median of study means was 8.1 years (IQR 4.4–14.7) (Table 2). The weighted average of stem subsidence across all studies was 2.5 mm and ranged from 0 to 10.4 mm. The majority of studies reported a small degree of subsidence, typically less than 3 mm [3, 6, 8, 18, 19, 29, 30, 32, 52]. Some studies reported cases of more severe subsidence, exceeding 10 mm in a few instances [36, 38, 53], and in isolated cases, subsidence reached up to 32 mm [2, 34].

**Table 2:** Graft incorporation rates

Author and year	Numbers of hips in study	Follow–up (mean, range)	Survivorship for aseptic loosening (%)	Cortical repair percentage	Trabecular incorporation percentage	Trabecular remodeling percentage	Overall graft incorporation percentage	Radiolucent lines observed (Y/N)	Radiolucent lines percentage	Radiolucent lines zones
Bunting et al. [3]	29	7.4 (0.7–13.9)	93% at 67 months	NA	NA	NA	NA	N	NA	NA
Iwase et al. [18]	99	11.0 (2.0–23.8)	87.1% at 10 years	NA	NA	NA	NA	N	NA	NA
Park et al. [32]	13	11.1 (5.3–15.1)	97.6% at 11.1 years	NA	NA	NA	NA	N	NA	NA
Stigbrand et al. [42]	69	7.0 (0.7–13.3)	93% at 10 years (95% CI: 86–100)	NA	NA	NA	NA	N	NA	NA
Wilson et al. [50]	705	14.7 (9.8–28.3)	98.8% at 20 years (aseptic loosening as endpoint)	NA	NA	NA	70%	Y	NA	NA
Wimmer et al. [51]	243	4.4 (NA)	93.8% after 8.8 years (95.7% for IBG; 93.1% for control)	NA	NA	NA	4.30%	Y	5.70%	NA
ten Have et al. [44]	31	12.6 (10.0–14.7)	77.4% at 11.6 years (95% CI: 9.6 to 13.5)	NA	NA	NA	88%	N	NA	NA
Howie et al. [16]	56	NA (2–15)	100%	NA	NA	NA	NA	N	NA	NA
Sierra et al. [38]	42	7.5 (NA)	90% at 5 and 10 years for stem revision	NA	NA	NA	NA	N	NA	NA
Wraighte and Howard [52]	75	10.5 (6.3–14.1)	92% at 10.5 years	NA			87%	Y	14.70%	NA
Ornstein et al. [29]	15	5.0 (NA)	100%	NA	20%	10%	20%	Y	NA	NA
Halliday et al. [13]	226	8.1 (5.0–16.0)	99.1% at 10 to 11 years for reoperation for symptomatic aseptic loosening	NA	28%	NA	28%	N	NA	NA
Gore et al. [12]	26	NA (0.0–6.0)	88.4% at 6 weeks	NA	NA	NA		N	NA	NA
Lind et al. [23]	87	3.6 (1.0–7.0)	96%	NA	NA	NA	88%	N	NA	NA
Robinson et al. [35]	57	2.3 (0.5–5.7)	Not specified	35.70%	39.30%	NA	35.70%	Y	NA	1
Biezen et al. [2]	21	5.0 (3.4–7.1)	100%	NA	33%	6%	50%	N	NA	NA
Pekkarinen et al. [34]	68	3.0 (0.1–6.0)	94.10%	NA	NA	NA	NA	N	NA	NA
Flugsrud et al. [10]	10	4.0 (3.0–4.6)	100%	NA	NA	NA	NA	N	NA	NA
Kärrholm et al. [19]	24	3.0 (NA)	100%	NA	NA	NA	NA	N	NA	NA
Eldridge et al. [8]	86	1.05 (0.5–2.8)	Not specified	NA	NA	NA	NA	N	NA	NA
Gie et al. [11]	56	2.5 (1.5–4.1)	100% at 24 months	NA	NA	NA	NA	N	NA	NA
Deakin and Bannister [6]	59	3.7 (0.5–11.0)	Not specified	NA	NA	NA	NA	Y	NA	NA
Edwards et al. [7]	70	3.1 (1.5–5.2)	98.5% at 37 months	NA	NA	NA	NA	Y	NA	NA
Knight and Helming [21]	31	2.6 (0.6–3.8)	Not specified	NA	NA	NA	NA	N	NA	NA
Mikhail et al. [28]	43	6.0 (5.0–7.0)	100% at 5 years	NA	NA	NA	NA	N	NA	NA
Oshima et al. [30]	55	7.7 (3.0–12.5)	Not specified	NA	31.60%	NA	31.60%	N	NA	NA
Schreurs et al. [37]	33	10.4 (8.0–13.0)	100% (one–sided 95% CI: 100% to 91.3%)	NA	NA	NA	NA	N	NA	NA
Singh and Bhalodiya [39]	53	5.5 (NA)	Not specified	48.90%	NA	63.80%	48.90%	N	NA	NA
Park et al. [33]	47	13.5 (10.9–17.8)	Not specified	NA	NA	NA	92%	Y	32%	NA
Te Stroet et al. [43]	37	9.0 (5.0–16.0)	100% (95% CI: 74.1–100%)	NA	NA	NA	NA	Y	NA	NA
Verspeek et al. [47]	33	17.0 (12.0–22.0)	Not specified	NA	NA	NA	NA	Y	NA	NA
Yan et al. [53]	15	7.7 (4.0–13.0)	Not specified	NA	NA	NA	NA	N	NA	NA

Based on prior IBG and RSA literature demonstrating front–loaded migration [11, 13, 19, 29], we defined the first postoperative year as the primary subsidence period. Because a minority of series report small additional settling beyond year 1 [3, 13, 33] and many studies cluster their reporting at 2–3 years [19, 29, 33] we report via a 3–year cutoff (≤ 3 vs > 3 years) to capture any late settling while maximizing comparability across studies. When stratifying by this cutoff and excluding the van Biezen outlier, studies with ≤ 3 years’ follow–up (n = 10) showed a pooled mean subsidence of 2.11 mm, whereas those with > 3 years (n = 9) showed 2.49 mm—a small absolute difference of 0.38 mm [2]. Taken together, these data support that most subsidence occurs within the first postoperative year, with only minor additional settling by ~ 3 years and minimal progression thereafter.

Retroversion of the stem was observed in 10 of 21 hips (48%) in one study using RSA, with retroversion angles reaching up to 5.3° in the most extreme case [19]. Varus alignment was reported in two studies, with 8 of 24 stems (33%) in one study showing varus alignment between 3° and 6° on postoperative radiographs, and 18 of 57 stems (32%) in another study placed in varus, with two stems progressing further during follow–up [2, 36]. Radiolucent lines were frequently reported during radiographic follow–up, commonly seen at the cement–graft interface or the graft–host interface. For example, one study [52] observed radiolucent lines in 14.7% of hips at the host–graft interface, while another [33] reported lines in 32% of hips in Gruen zones.

### Graft incorporation

Graft incorporation rates, including cortical repair and trabecular incorporation, were variable across the studies (Table 3). All studies used conventional radiographs for assessments of graft incorporation. These evaluations focused on indicators such as cortical repair, trabecular incorporation, and the presence of radiolucent lines at the graft–host or cement–graft interfaces. Studies relied on classifications such as the Gruen zones, DeLee and Charnley classification, and even dual–energy X–ray absorptiometry scanning for more detailed assessment of bone mineral density and healing (Table 1).

**Table 3:** Subsidence rates

Author and year	Numbers of hips in study	Follow–up (mean, range)	Subsidence (Range)	Time point at which subsidence was evaluated (years)	Stem migration (besides subsidence)
Bunting et al. [3]	29	7.4 (0.7–13.9)	1.89 (NA)	5	Not evaluated
Iwase et al. [18]	99	11.0 (2.0–23.8)	1.5 (NA)	11	Not evaluated
Park et al. [32]	13	11.1 (5.3–15.1)	0.67 (0.05–2.81)	11.1	Not evaluated
Stigbrand et al. [42]	69	7.0 (0.7–13.3)	0 (NA)	7	Not evaluated
Wilson et al. [50]	705	14.7 (9.8–28.3)	NA (NA)	14.7	Not evaluated
Wimmer et al. [51]	243	4.4 (NA)	NA (NA)	4.4	Not evaluated
Ten Have et al. [44]	31	12.6 (10.0–14.7)	NA (NA)	12.6	Not evaluated
Howie et al. [16]	56	NA (2.0–15.0)	NA (NA)	1	Not evaluated
Sierra et al. [38]	42	7.5 (NA)	5.6 (1–17)	7.5	Not evaluated
Wraighte and Howard [52]	75	10.5 (6.3–14.1)	2 (1–4)	1	No
Ornstein et al. [29]	15	5.0 (NA)	2.9 (2.9–2.9)	5	Yes
Halliday et al. [13]	226	8.1 (5.0–16.0)	3 (3–3)	1	Not evaluated
Gore et al. [12]	26	NA (0.0–6.0)	6 (6–6)	Exact timepoint not specified	Not evaluated
Lind et al. [23]	87	3.6 (1.0–7.0)	NA (NA)	3.6	No
Robinson et al. [35]	57	2.3 (0.5–5.67)	4.1 (0.38–12.5)	2.25	Yes
Biezen et al. [2]	21	5.0 (3.42–7.08)	10.38 (0–32)	1	Yes
Pekkarinen et al. [34]	68	3.0 (0.1–6)	3 (0–36)	1	Yes
Flugsrud et al. [10]	10	4.0 (3.0–4.6)	2 (2–5)	2	Not evaluated
Kärrholm et al. [19]	24	3.0 (NA)	0.32 (–2.0–0.31)	2	No
Eldridge et al. [8]	86	1.05 (0.5–2.8)	0 (0–0)	2	No
Gie et al. [11]	56	2.5 (1.5–4.08)	3 (NA)	2	Not evaluated
Deakin and Bannister [6]	59	3.7 (0.5–11.0)	1.28 (NA)	3.7	Not evaluated
Edwards et al. [7]	70	3.1 (1.5–5.2)	NA (NA)	3.1	Yes
Knight and Helming [21]	31	2.6 (0.6–3.8)	NA (NA)	0.5	Yes
Mikhail et al. [28]	43	6.0 (5.0–7.0)	2–4 (2–4)	1	Not evaluated
Oshima et al. [30]	55	7.7 (3.0–12.5)	0.7 (0–3)	1	Not evaluated
Schreurs et al. [37]	33	10.4 (8.0–13.0)	3 (NA)	Exact timepoint not specified	No
Singh and Bhalodiya [39]	53	5.5 (NA)	NA (NA)	Exact timepoint not specified	Not evaluated
Park et al. [33]	47	13.5 (10.9–17.8)	NA (NA)	3.2	Not evaluated
te Stroet et al. [43]	37	9.0 (5.0–16.0)	NA (NA)	9	No
Verspeek et al. [47]	33	17.0 (12.0–22.0)	2.9 (2.9–2.9)	15	No
Yan et al. [53]	15	7.7 (4.0–13.0)	5.7 (0.6–17)	7.7	No

Across the studies, radiographic evidence of graft incorporation generally appears within the first postoperative year. Cortical repair was detected as early as 6–7 months postoperatively, with some series reporting an average onset at 7 months and cortical healing progressing for an average of 37 months [13, 30, 33]. Trabecular incorporation is first noted on radiographs between 3 and 6 months postoperatively, and in some studies, trabecular remodeling was observed in up to 21–50% of evaluated Gruen zones at midterm follow–up [7, 13, 29]. Overall graft incorporation percentages were reported to be high, with several investigators documenting incorporation rates of approximately 80% to 90% or greater by 12 months postoperatively [6, 7, 13]. Radiolucent lines were first noted on routine follow–up radiographs in the early postoperative period, typically within 3 to 6 months, most frequently appearing in Gruen zones 1 and 7; the reported radiolucent line percentages varied among studies but were generally low (< 10–15% of hips or zones) and the lines were characteristically thin (< 2 mm) [29, 33, 52]. Across the published series, no consistent correlation was identified between the presence or extent of radiolucent lines and either the magnitude of stem subsidence or the need for re–revision; in several studies, small, nonprogressive radiolucent lines did not predict increased subsidence or subsequent re–revision [7, 13, 33].

### Meta–analyses

**Fig. 2:**
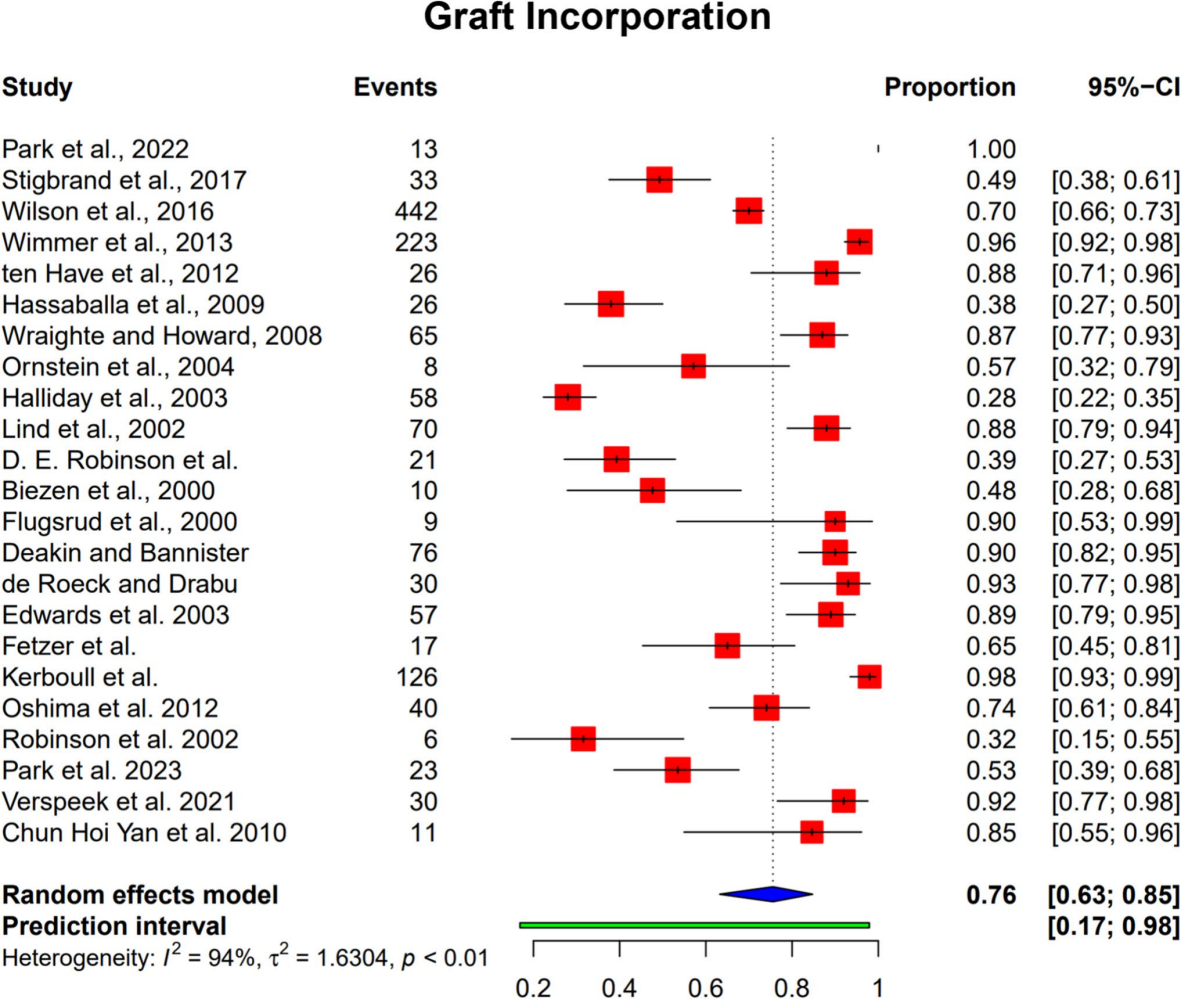
Pooled graft incorporation rates. The forest plot shows the proportion of surviving grafts with the size of squares representing the weight of each study. I2 represents heterogeneity

The graft incorporation rates across 21 studies are summarized in Fig. 2. The pooled proportion of graft incorporation was 76% (95% CI: 63%–85%), indicating generally favorable outcomes. However, there was high heterogeneity among the included studies (I^2^ = 94%, *p* < 0.01).

**Fig. 3:**
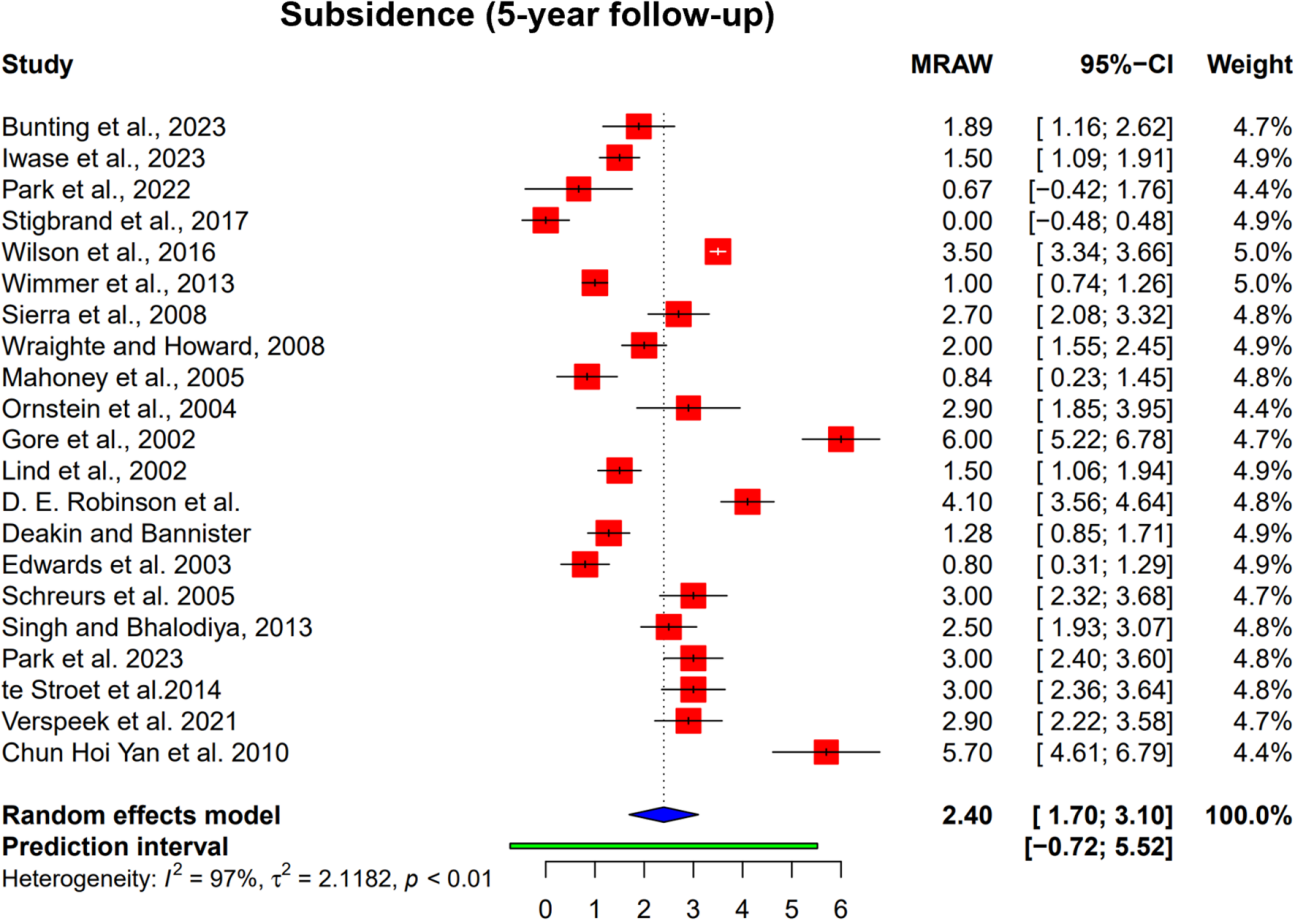
Pooled subsidence rates at 5 years. The forest plot shows the proportion of surviving grafts with the size of squares representing the weight of each study. I2 represents heterogeneity

Similarly, the pooled stem subsidence values for studies with a minimum 5–year follow–up were 2.40 mm (95% CI 1.70–3.10 mm), as illustrated in Fig. 3. Heterogeneity for this result was high (I^2^ = 97%). Subgroup analysis (Table 4) showed that fresh–frozen allografts tended to have a higher incorporation rate (79.0% [95% CI 62.7%–89.4%]) than processed allografts (69.9% [95% CI 45.7%–86.5%]). However, this difference was not statistically significant (*p* = 0.399).

**Table 4:** Subgroup analysis for graft incorporation

	Number of Studies	Proportion	95% Confidence Interval	*p*–value
*Graft type*				0.399
Allograft	9	0.699	0.457–0.865	
Fresh–frozen allograft	14	0.790	0.627–0.894	
*Age*				0.742
≥ 65	14	0.770	0.625–0.871	
< 65	9	0.731	0.429–0.907	
*Follow–up*				0.560
≥ 5 years	14	0.728	0.562–0.848	
< 5 years	9	0.792	0.542–0.925	
*Endo–Klinik*				0.263
Grades I + II	5	0.550	0.092–0.936	
Grades III + IV	10	0.778	0.639–0.875	

Age did not significantly affect graft incorporation rates; patients aged ≥ 65 years had an incorporation rate of 77.0% (95% CI 62.5%–87.1%), while those under 65 years had a rate of 73.1% (95% CI 42.9%–90.7%) (*p* = 0.742).

Follow–up duration also did not show a significant impact. Studies with a follow–up of ≥ 5 years reported an incorporation rate of 72.8% (95% CI 56.2%–84.8%), whereas those with less than 5 years had a rate of 79.2% (95% CI 54.2%–92.5%) (*p* = 0.560). Patients with severe bone loss (Endo–Klinik grades III and IV) tended to have a higher incorporation rate (77.8% [95% CI 63.9%–87.5%]) compared to those with less severe bone loss (grades I and II) (55.0% [95% CI 9.2%–93.6%]), although this difference did not reach statistical significance (*p* = 0.263).

Subgroup analysis for stem subsidence performed on studies with at least 5 years of follow–up is presented in Table 5. Gender differences were observed but were not statistically significant (*p* = 0.181); females: 2.82 mm (95% CI 1.66–3.98 mm); males: 1.94 mm (95% CI: 1.05–2.83 mm).

**Table 5:** Subgroup analysis for stem subsidence at 5 or more years follow–up

	Number of studies	Mean (mm)	95% CI (mm)	*p*–value
*Gender*				0.181
Male	10	1.943	1.054–2.832	
Female	11	2.816	1.655–3.977	
*Graft Type*				0.460
Autograft	2	2.270	– 1.485–6.026	
Processed Allograft	5	3.253	0.959–5.548	
Fresh–frozen allograft	14	2.117	1.256–2.977	
*Cemented*				0.358
Yes	19	2.471	1.703–3.239	
No	2	1.706	– 7.807–11.219	
*Age*				0.301
≥ 65	12	2.107	1.083–3.131	
< 65	9	2.797	1.698–3.895	
*Follow–up*				0.077
≥ 5 years	15	2.735	1.871–3.600	
< 5 years	6	1.582	0.267–2.896	
*Paprosky*				0.486
Grades I + II	3	1.064	0.229–1.898	
Grades III + IV	2	1.710	– 9.807–13.227	
*Endo–Klinik*				0.634
Grades I + II	3	2.781	– 0.777–6.338	
Grades III + IV	8	2.294	0.873–3.714	

Fresh–frozen allografts tended to show lower mean subsidence (2.12 mm, 95% CI 1.26–2.98 mm) compared to processed allografts (3.25 mm, 95% CI 0.96–5.55 mm), although this difference was not statistically significant (*p* = 0.460). Cemented stems demonstrated a tendency toward higher subsidence (2.47 mm, 95% CI 1.70–3.24 mm) relative to uncemented stems (1.71 mm, 95% CI − 7.81–11.22 mm), but the difference was not statistically significant (*p* = 0.358).

Patients under 65 years tended to have higher subsidence (2.80 mm, 95% CI 1.70–3.90 mm) compared to those aged ≥ 65 years (2.11 mm, 95% CI 1.08–3.13 mm), although this was not statistically significant (*p* = 0.301). A trend toward greater subsidence with longer follow–up was observed. Studies with follow–up ≥ 5 years reported subsidence of 2.73 mm (95% CI 1.87–3.60 mm) compared to 1.58 mm (95% CI 0.27–2.90 mm) in studies with less than 5 years, but this was not statistically significant (*p* = 0.077).

## Discussion

Revision THA with femoral IBG demonstrates a pooled graft incorporation rate of 76% (95% CI, 63%–85%). Weighted average stem subsidence was 2.5 mm (95% CI, 1.7–3.1 mm), with the majority of this subsidence occurring in the first year and subsequently stabilizing. Notably, none of the evaluated risk factors—including patient age, graft type, and the severity of preoperative bone loss—were significantly associated with these outcomes (*p* > 0.05). Defining failure in femoral IBG is challenging because stems may remain clinically stable even when subsidence and radiolucent lines are observed during follow–up. Some authors consider stem subsidence greater than 5 mm as construct failure [8, 9], but others report that subsidence does not necessarily correlate with poor clinical outcomes [46, 53]. Edwards et al. found moderate–to–massive subsidence in 19% of femora without resulting in stem loosening [7]. Yan et al. observed that subsidence did not adversely affect the Harris Hip Score, thigh pain, or aseptic loosening, indicating that radiographically confirmed axial migration does not preclude favorable clinical outcomes [53]. Therefore, in this review we predefined ≥ 5 mm as clinically relevant and ≥ 10 mm as massive subsidence for interpretive context.

Graft integration typically initiates within the first 6–12 months postoperatively Oshima et al. [30]. reported early signs of cortical repair at an average of 7 months, with remodeling continuing for up to six years. Similarly, Singh and Bhalodiya [39] observed signs of graft osteointegration between 9 and 18 months, while Deakin and Bannister [6] documented trabeculation and cortical repair in 90–96% of cases within the first postoperative year. Park et al. noted cortical repair and trabecular remodeling in nearly half and two–thirds of cases, respectively, and observed stabilization of stem subsidence within 1–2 years [33]. Collectively, these findings indicate that cortical repair may precede trabecular remodeling, and both typically occur within the first postoperative year.

In this review, the mean subsidence was 2.5 mm, and most studies indicated that the majority of subsidence occurred within the first postoperative year, with minor additional settling through ~ 3 years and subsequent stabilization. Moderate subsidence (≤ 5 mm) was not associated with increased rates of re–revision. For example, Deakin and Bannister reported five cases with > 5 mm subsidence, but only one revision, which was due to instability rather than loosening [6]. Similarly, Singh and Bhalodiya documented two cases of > 10 mm subsidence with no subsequent stem–related revisions [39]. Edwards et al. observed one case of > 10 mm subsidence in 38 hips (2.6%), which did not result in mechanical failure [7].

Massive stem subsidence (> 10 mm) is rare and does not consistently predict the risk of component–related re–revision. Singh and Bhalodiya reported two hips (4.2%) with subsidence exceeding 10 mm, but none required re–revision [39]. Similarly, Park et al. reported one case (7.7%) of subsidence exceeding 10 mm, again without the need for reoperation [32]. Deakin and Bannister observed five hips with subsidence greater than 5 mm—including one case of 17 mm—yet only one required revision due to instability [6]. In Edwards et al., only a single hip out of 38 (2.6%) experienced subsidence over 10 mm, which was the only major femoral failure in the cohort [7]. These examples highlight that while isolated cases of massive early subsidence may raise concerns for mechanical compromise, the overall incidence of re–revision in such cases remains low. This trend suggests that massive subsidence alone—particularly when not progressive—may not reliably predict clinical failure, and should instead be interpreted alongside other signs such as progressive migration or the development of radiolucent lines.

Within this context, each stem design has its own inherent subsidence pattern—throughout our review, we found studies that support and challenge the traditional benefit associated with controlled subsidence. Lamberton et al. observed that subsidence with the Exeter stem is expected and beneficial when it occurs early and stabilizes thereafter [22]. Gie et al. [11] reported favorable outcomes with the polished, tapered, collarless Exeter stem, where controlled subsidence averaged about 3 mm in the first 13 months before stabilizing. This controlled movement was associated with successful graft incorporation, evidenced by trabecular remodeling and cortical repair in 70% of cases. Halliday et al. [13] noted minimal subsidence with the Exeter stem, averaging 3 mm at one year, attributing this to the polished surface and double–tapered design that facilitates controlled subsidence and graft compression. Their study reported high rates of graft incorporation, with trabecular remodeling in 34% of cases and cortical healing in 87%.

Mahoney et al. found a mean subsidence of 0.84 mm with collarless, polished, tapered stems over 4.7 years [24]. Knight and Helming reported subsidence in 15 of 30 patients using the collarless polished tapered stem, mostly occurring within the first six months and then stabilizing, attributing this to the stem design that permits controlled subsidence [21]. Their study noted effective graft incorporation, with trabecular remodeling in 50% of patients and cortical repair in 33%.

In our review, 40% of the studies reported no stem migration or subsidence, while others recorded minor subsidence, typically less than 3 mm (Table 2), without affecting graft incorporation rates (Table 3). These findings suggest that with certain stem designs and proper surgical technique, controlled subsidence does not impair graft incorporation. This raises the question of whether controlled subsidence or greater initial stability without subsidence is preferable. Kerboull et al. [20] emphasized that significant subsidence is unnecessary with the Charnley–Kerboull stem and may indicate failure. Their study, along with findings from Stigbrand et al. [42] using the matte, collared Lubinus SP II stem, demonstrated successful graft incorporation without relying on stem subsidence. Specifically, they reported generalized healing with trabecular formation in 33 cases and cortical repair in 22 cases, indicating that graft incorporation can be achieved through immediate axial support and frictional stability afforded by specific stem designs. Wimmer et al. [51] employed an uncemented, conical, tapered titanium stem (MRP–TITAN) with minimal subsidence (95.7% of patients showed no stem migration). They reported incomplete bony union in only 4.3% of cases, suggesting that successful graft incorporation can occur with the use of cementless stems relying on early stability and biologic fixation.

Kerboull et al. [20] emphasized that significant subsidence is unnecessary with the Charnley–Kerboull stem and may indicate failure. Their study, along with findings from Stigbrand et al. [42] using the matte, collared Lubinus SP II stem, demonstrated successful graft incorporation without relying on stem subsidence. Specifically, they reported generalized healing with trabecular formation in 33 cases and cortical repair in 22 cases, indicating that graft incorporation can be achieved through immediate axial support and frictional stability afforded by specific stem designs. Wimmer et al. [51] employed an uncemented, conical, tapered titanium stem (MRP–TITAN) with minimal subsidence (95.7% of patients showed no stem migration). They reported incomplete bony union in only 4.3% of cases, suggesting that successful graft incorporation can occur with the use of cementless stems relying on early stability and biologic fixation. Although successful graft incorporation stabilizes the graft–host interface, reducing micromotion and further subsidence, the interaction between these processes over time remains unclear. Our review suggests that early stem migration may stimulate graft remodeling through mechanical loading, potentially enhancing incorporation and vice versa. Achieving an optimal balance of mechanical stability and biological activity appears crucial [5]. Controlled early migration within acceptable limits might be beneficial for graft incorporation, supporting the concept that mechanical loading stimulates biological activity [34]. Understanding this balance could inform surgical techniques and postoperative rehabilitation (e.g., early mobilization with protected weight–bearing), optimizing both mechanical and biological outcomes.

Severe preoperative bone loss has been associated with increased stem subsidence in femoral IBG. Preoperative bone loss was reported in 79% of studies (26/33), with severe bone loss (Grades III and IV) noted in 60% of cases using the Paprosky system and 55% using the Endo–Klinik classification (Table 1). Several studies observed higher incidence of substantial subsidence during the early postoperative period in patients with severe preoperative bone loss [2, 13, 53]. Our subgroup analysis showed that patients with Endo–Klinik grades III and IV had a mean subsidence of 3.92 mm at two years, compared to 2.57 mm in grades I and II, although this was not statistically significant (*p* = 0.331) (Table 4).

Longer standard stems (> 200 mm) may bypass areas of poor bone quality, providing better initial stability [3]. In our review, long stems were used in 30% of studies, typically in cases with significant bone loss (Table 1). While our subgroup analysis did not find a statistically significant difference in subsidence between short and long stems at two years (*p* = 0.632) (Table 4), the use of longer stems may still be beneficial in certain clinical scenarios. It is possible that surgeons selected longer stems primarily for more challenging defects, introducing a degree of selection bias that may have obscured or minimized any true difference in subsidence outcomes related to stem length.

Fresh–frozen femoral head allograft was the most commonly used bone graft material, reported in 73% of the studies (24/33) (Table 1). The use of fresh–frozen allografts resulted in higher subsidence at two years (3.52 mm) compared to autografts (1.36 mm), although this difference was not statistically significant (*p* = 0.104) (Table 4). Robinson et al. reported that the use of irradiated bone grafts was associated with a lack of trabecular remodeling and higher subsidence rates [35]. Our analysis also indicated that cemented stems were associated with a higher mean subsidence of 2.47 mm (95% CI 1.70–3.24 mm) compared to uncemented stems, which had a mean subsidence of 1.71 mm (95% CI − 7.81–11.22 mm). However, this difference was not statistically significant (*p* = 0.358) (Table 5).

Varus stem positioning has been associated with increased subsidence due to uneven stress distribution and micromotion at the graft–cement interface. Gore et al. highlighted technical errors like varus placement leading to increased subsidence [12]. Proper alignment is crucial to ensure even load distribution and minimize micromotion [1]. Meticulous surgical technique is essential for achieving stable graft impaction, as inadequate impaction can result in graft settling and stem subsidence. Gore et al. attributed complications to inadequate graft packing and failure to provide sufficient medial support [12]. Yan et al. noted difficulties in achieving satisfactory graft impaction in cases with severe bone loss [53]. Reinforcing cortical defects with strut grafts or meshes can provide additional support, reducing stress on the graft and preventing subsidence [29, 38]. An inadequate cement mantle may compromise stem fixation, leading to micromotion and subsidence. Masterson et al. emphasized the importance of ensuring a uniform and adequate cement mantle—tailored to the specific design implant—is critical in preventing stem migration [25].

While axial migration is a common focus in evaluating femoral stem performance, migration can occur in multiple directions, including rotational movements such as varus–valgus tilt and anteversion–retroversion, which can be detected via radiostereometric analysis (RSA). Bunting et al. conducted an RSA study on patients undergoing staged revision THA with femoral IBG [3]. At two years, the median stem subsidence relative to the femur was − 1.36 mm (range: –0.31 mm to− 4.98 mm), and at five years, it was − 1.89 mm (range: − 0.27 mm to − 6.35 mm). They observed continued median stem subsidence between two and five years of − 0.65 mm (range: + 0.5 mm to − 1.9 mm). The study noted that most subsidence occurred within the first two years postoperatively and then stabilized, aligning with our literature review findings that significant further subsidence is rare after 12 months.

Bunting et al. also reported minimal rotational movements, indicating good implant stability in multiple planes [3]. This stability suggests successful graft incorporation and supports the notion that proper surgical technique and implant selection can mitigate multidirectional migration. Howie et al. emphasized the importance of RSA in detecting early stem migration and rotational movements, which are predictive of long–term outcomes [16]. They found that stems demonstrating minimal early migration had better clinical results and survivorship. Rotational stability is as crucial as axial stability, especially in the context of IBG where graft remodeling and incorporation play significant roles in achieving long–term fixation. Early identification of multidirectional migration can allow for timely intervention, adjusting rehabilitation protocols or planning revisions before clinical failure occurs.

This review has several limitations. The included studies displayed significant heterogeneity in patient selection, stem designs, graft materials, and surgical techniques. Variability in reporting outcomes, especially regarding stem subsidence and graft incorporation, made it difficult to conduct a uniform analysis. Different thresholds were used to define significant subsidence—with some studies classifying ≥ 5 mm as clinically meaningful, while others considered ≥ 15 mm to be “massive” subsidence—creating inconsistency in reporting and complicating direct comparisons. Standard radiographs, while widely used to assess graft integration, are limited in their ability to capture the complexity of a three–dimensional biological process. Overlapping structures, low resolution, and subjective interpretation may obscure signs of trabecular remodeling or cortical repair, delaying recognition of true biologic incorporation and reducing reproducibility across studies. Many studies were retrospective with relatively small sample sizes, which may limit the strength of the conclusions. Additionally, the absence of randomized controlled trials and standardized measurement methods across studies may introduce bias and affect the reliability of the results.

## Conclusion

Femoral IBG for rTHA demonstrates variable graft incorporation rates, averaging 76% (95% CI: 63%—85%). Stem subsidence averaged 2.5 mm (95% CI: 1.7 mm–3.1 mm) at mid–to–long–term follow–up. The variability in stem subsidence and graft incorporation was not significantly influenced by bone graft type, stem design, age or preoperative bone loss.

## Data Availability

No datasets were generated or analysed during the current study.
